# Supplementary material for: Comparative genomics provides new insights into the diversity, physiology, and sexuality of the only industrially exploited tremellomycete: Phaffia rhodozyma
Source: BMC Genomics. 2016 Nov 9;17:901. doi: 10.1186/s12864-016-3244-7 (PMC5103461; doi:10.1186/s12864-016-3244-7)
Supplement: Additional file 6: — List of orphan genes with links to PFAM (related to Additional file 1: Table S1). (ZIP 1428 kb) [file 12864_2016_3244_MOESM6_ESM.zip › PFAM_UNIQUE/G04363_P.html]

#                                                                            --- full sequence --- -------------- this domain -------------   hmm coord   ali coord   env coord
  
# target name        accession   tlen query name           accession   qlen   E-value  score  bias   #  of  c-Evalue  i-Evalue  score  bias  from    to  from    to  from    to  acc description of target
  
#------------------- ---------- ----- -------------------- ---------- ----- --------- ------ ----- --- --- --------- --------- ------ ----- ----- ----- ----- ----- ----- ----- ---- ---------------------
  
G04363\_P             -            217 DUF4045              PF13254.1    418      0.43    8.5   4.1   1   1    0.0017      0.54    8.1   4.1    43   111    82   152    69   183 0.63 - Domain of unknown function (DUF4045)  
G04363\_P             -            217 Gp37\_Gp68            PF07505.6    261     0.038   11.8   0.0   1   1   7.2e-06     0.043   11.6   0.0   160   209   158   208    36   211 0.76 - Phage protein Gp37/Gp68  
G04363\_P             -            217 Myc\_N                PF01056.13   329      0.27    9.0   2.3   1   1    0.0015      0.35    8.7   2.3   205   257   104   155    74   181 0.59 - Myc amino-terminal region  
G04363\_P             -            217 Presenilin           PF01080.12   403       0.2    9.1   1.8   1   1   0.00021      0.25    8.8   1.8   197   291    61   160    59   187 0.56 - Presenilin
